# Supplementary material for: Content-rich biological network constructed by mining PubMed abstracts
Source: BMC Bioinformatics. 2004 Oct 8;5:147. doi: 10.1186/1471-2105-5-147 (PMC528731; doi:10.1186/1471-2105-5-147)
Supplement: Additional File 2 — The original results of the above study (non-essential files are deleted to keep the file size under the limit set by BMC bioinformatics). [file 1471-2105-5-147-S2.bz2 › chilibotAdditionalFile2/dip40/656ID10449718E11047/html/TRPE_TRPG.html]

 


 **TRPE** and **TRPG** 
  
Found 35 abstracts in PubMed, retrieved 35.  
 

 What does Google say? 
 PDF only 
| .edu only 

---

**Interactive relationship** (e.g. stimulation, inhibition, etc)

**Neutral relationship**- This is in contrast to L. biflexa, in which  **trpE**  is separated from  **trpG**  by only 64 bp.  Ref: 1987149 J Bacteriol, 1991
- Constructions with the regulatory signals coupled to the wt  **trpE**   **trpG**  genes were introduced in a B. lactofermentum  **trpE**  mutant obtained by gene disruption.  Ref: 7510262 Gene, 1994
- It is a heterotetramer of anthranilate synthase  **TrpE**  and glutamine amidotransferase  **TrpG**  subunits, in which two  **TrpG**   **TrpE**  protomers associate mainly via the  **TrpG**  subunits.  Ref: 10449718 Proc Natl Acad Sci U S A, 1999
- ... Pseudomonas  **TrpE**  and  **TrpG**  are more closely related to E. coli PabB and PabA than to E. coli  **TrpE**  and  **TrpG** .  Ref: 2153661 J Bacteriol, 1990
- The  **trpE**  gene overlapped at the termination codon with a putative initiation codon of  **trpG**  trp G D, as deduced from the amino acid sequence homology with anthranilate synthase II of Serratia marcescens.  Ref: 2732211 J Biochem Tokyo , 1989
- The Thermotoga maritima trp operon is arranged with  **trpE**  forming an overlapping transcript with a second protein consisting of a fusion of anthranilate synthase component II  [ **TRPG** ]   **trpG**  and anthranilate phosphoribosyltransferse trpD.  Ref: 7685830 J Mol Biol, 1993
- The structure suggests a model in which chorismate binding triggers a relative movement of the two domain tips of the  **TrpE**  subunit, activating the  **TrpG**  subunit and creating a channel for passage of ammonia toward the active site of the  **TrpE**  subunit.  Ref: 10449718 Proc Natl Acad Sci U S A, 1999
- The  **trpE**  gene was preceded by an attenuator like structure and followed by the  **trpG**  gene, with a short gap between them.  Ref: 2844259 Biochim Biophys Acta, 1988

**Non-interactive relationship** (e.g. studied together, co-existance, homology, etc.)

- Surprisingly, PhnA and PhnB are more closely related to E. coli  **TrpE**  and  **TrpG**  than to Pseudomonas  **TrpE**  and  **TrpG** .  Ref: 2153661 J Bacteriol, 1990
- Phylogenetic analyses were performed using  **trpE**  and  **trpG**  from the endosymbionts of these four aphids as well as from the endosymbiont of Schlechtendalia chinensis, in which trpEG occurs on the chromosome.  Ref: 8642610 J Mol Evol, 1996
- A systematic polymerase chain reaction PCR analysis of P. aeruginosa strains that produce various subtypes of R and F pyocins revealed that the genes for every subtype are located between  **trpE**  and  **trpG**  in the same or very similar gene organization as for R2 and F2 pyocins, but with alterations in genes that determine the receptor specificity.  Ref: 11069649 Mol Microbiol, 2000
- The results indicated that  **trpE**  and  **trpG**  are clustered in S. venezuelae.  Ref: 2040427 FEMS Microbiol Lett, 1991
- This apparent gene fusion joins the C terminus of the large subunit  **TrpE**  to the N terminus of the small subunit  **TrpG**  through a short connecting segment.  Ref: 2656657 J Bacteriol, 1989
- These rules explain known  **trpG**  like fusions and explain why  **trpG**  and pabA are not fused to  **trpE**  and pabB, respectively.  Ref: 2982857 J Biol Chem, 1985
- Following the nomenclature established for E. coli and Serratia marcescens, the B. lactofermentum trp genes were designated trpL,  **trpE** ,  **trpG** , trpD, trpC including the trpF domain, trpB, and trpA.  Ref: 2823076 Mol Gen Genet, 1987
- The estA gene showed high similarity to an open reading frame of unknown function located in the  **trpE**   **trpG**  region of P. putida and to a gene encoding an outer membrane esterase of Salmonella typhimurium.  Ref: 10559163 J Bacteriol, 1999
- This report describes the cloning and sequencing of the tryptophan related gene pair, designated  **trpE**  and  **trpG** , and presents experiments implicating their gene products in tryptophan production.  Ref: 2105306 J Bacteriol, 1990
- Amino acid sequence comparison with 19  **trpE** , 18  **trpG**  and 14 trpD genes from other organisms suggest that the Thermotoga trp genes resemble corresponding genes from other thermophiles more closely than expected.  Ref: 7685830 J Mol Biol, 1993
- Tk  **trpE**  and Tk  **trpG** , the genes that encode the two subunits of anthranilate synthase from the hyperthermophilic archaeon Thermococcus kodakaraensis KOD1, have been expressed independently in Escherichia coli.  Ref: 11237738 Biochem Biophys Res Commun, 2001
- Expression of Sulfolobus solfataricus  **trpE**  and  **trpG**  genes in E. coli.  Ref: 9016772 Biochem Biophys Res Commun, 1997
- The plasmid population was heterogeneous with respect to the number of tandem repeats of a 1.8 kb unit carrying repAC1,  **trpG** , and remnants of  **trpE** .  Ref: 9872768 Appl Environ Microbiol, 1999
- Nucleotide sequences upstream of  **trpE**  and  **trpG**  encode putative leader peptides similar in sequence to leader peptides found in other bacterial species, and the phenotypes of the mutants strongly suggest that transcription of these operons is regulated solely by attenuation.  Ref: 11325956 J Bacteriol, 2001
- We have determined the DNA sequence of the distal 148 codons of  **trpE**  and all of  **trpG**  in Pseudomonas aeruginosa.  Ref: 3127654 Mol Biol Evol, 1986
- The products of  **trpE** ,  **trpG** , and trpC from S. solfataricus were compared to the homologous products from a eukaryote, a eubacterium, and two archaebacteria, namely, a methanogen and an extreme halophile.  Ref: 8416906 J Bacteriol, 1993
- The nucleotide sequence of a 2 4 kb BamHI SalI fragment of Streptomyces venezuelae ISP5230 DNA that complements  **trpE**  and  **trpG**  mutations in Escherichia coli contains two ORFs.  Ref: 9695930 Microbiology, 1998
- its genes are designated  **trpE**  and  **trpG** .  Ref: 2153661 J Bacteriol, 1990
- The nucleotide sequence of the region of the L. biflexa serovar patoc chromosome encoding the  **trpE**  and  **trpG**  genes has been determined.  Ref: 2703466 J Bacteriol, 1989
- We have confirmed this observation and have shown that expression of two reporter gene fusions,  **trpE**  lacZ and  **trpG**  lacZ, is also increased under these conditions.  Ref: 8932308 J Bacteriol, 1996
- The anthranilate synthase complex Tk AS complex was obtained by heat treatment of the mixture of cell free extracts containing each recombinant protein, Tk  **TrpE**  alpha subunit and Tk  **TrpG**  beta subunit, at 85 degrees C for 10 min.  Ref: 11237738 Biochem Biophys Res Commun, 2001
- Three pairs of genes  **trpG**  trpD, trpC trpF, and trpB trpA overlap, and there is intercistronic spacing of 12 6, and 585 bp between the  **trpE**   **trpG** , trpD trpC, and trpF trpB gene pairs, respectively.  Ref: 1400208 J Bacteriol, 1992
- The genes for the large and small subunits of anthranilate synthase  **trpE**  and  **trpG** , respectively have been cloned from Pseudomonas aeruginosa PAC174 into E. coli by R prime formation with the broad host range plasmid R68.44.  Ref: 3127655 Mol Biol Evol, 1986
- The wt and mutated OP regions were coupled to the amy reporter gene encoding alpha amylase Amy or to the 5 region  **trpE**  and  **trpG**  genes of the trp operon, for expression studies.  Ref: 7510262 Gene, 1994
- The genes  **trpE**  and  **trpG**  of the hyperthermophilic archaeon Sulfolobus solfataricus, encoding the components I and II of anthranilate synthase, were cloned and co expressed in Escherichia coli.  Ref: 9016772 Biochem Biophys Res Commun, 1997
- Analysis of the predicted amino acid sequences of the proteins encoded by the ORFs allowed us to identify the  **trpE**  and  **trpG**  genes of L. biflexa.  Ref: 2703466 J Bacteriol, 1989
- In bacteria, the first reaction of the tryptophan biosynthetic pathway involves the conversion of chorismate and glutamine to anthranilate by the action of anthranilate synthase, which is composed of the alpha  **trpE**  gene product and beta  **trpG**  gene product subunits.  Ref: 12634337 Microbiology, 2003
- This report describes the cloning and sequencing of P. putida  **trpE** ,  **trpG** , trpD, and trpC.  Ref: 2404959 J Bacteriol, 1990
- **trpE**  and  **trpG**  are independently transcribed.  Ref: 2153661 J Bacteriol, 1990
- Identification and nucleotide sequence of the Leptospira biflexa serovar patoc  **trpE**  and  **trpG**  genes.  Ref: 2703466 J Bacteriol, 1989
- Transformation of E. coli ED23 lacking both  **trpE**  and  **trpG**  functions with plasmids containing the 2.4 kb DNA segment gave prototrophic strains exhibiting both the ASI and ASII activities of anthranilate synthetase.  Ref: 2040427 FEMS Microbiol Lett, 1991
- The rate limiting enzyme in tryptophan biosynthes, anthranilate synthase, is encoded by the genes  **trpE**  and  **trpG** .  Ref: 9299222 Mol Phylogenet Evol, 1997
- Comparison of the amino acid sequences of the  **trpE**  gene product and the  **trpG**  gene product with corresponding gene products from other bacteria showed regions of highly conserved sequence.  Ref: 2703466 J Bacteriol, 1989
- Introduction of an inactivated  **trpE**  gene into a phnA mutant abolished residual pyocyanin production, suggesting that the  **trpE**   **trpG**  gene products are capable of providing some anthranilate for pyocyanin synthesis.  Ref: 2153661 J Bacteriol, 1990
- The  **TrpE** ,  **TrpG** , TrpC, TrpA, and TrpD proteins were 30 to 50% identical to those from representatives of other species.  Ref: 1860817 J Bacteriol, 1991
- Molecular cloning and nucleotide sequence of Thermus thermophilus HB8  **trpE**  and  **trpG** .  Ref: 2844259 Biochim Biophys Acta, 1988
